# A quantitative content analysis of UK newsprint coverage of proposed legislation to prohibit smoking in private vehicles carrying children

**DOI:** 10.1186/s12889-015-2110-x

**Published:** 2015-08-08

**Authors:** Chris Patterson, Sean Semple, Karen Wood, Sheila Duffy, Shona Hilton

**Affiliations:** MRC/CSO Social and Public Health Sciences Unit, University of Glasgow, 200 Renfield Street, Glasgow, G2 3QB UK; Division of Applied Health Sciences, Scottish Centre for Indoor Air, University of Aberdeen, Aberdeen, AB25 2ZD UK; Action on Smoking & Health (Scotland), 8 Frederick Street, Edinburgh, EH2 2HB UK

## Abstract

**Background:**

Mass media representations of health issues influence public perceptions of those issues. Despite legislation prohibiting smoking in public spaces, second-hand smoke (SHS) remains a health risk in the United Kingdom (UK). Further legislation might further limit children’s exposure to SHS by prohibiting smoking in private vehicles carrying children. This research was designed to determine how UK national newspapers represented the debate around proposed legislation to prohibit smoking in private vehicles carrying children.

**Methods:**

Quantitative analysis of the manifest content of 422 articles about children and SHS published in UK and Scottish newspapers between 1st January 2003 and 16th February 2014. Researchers developed a coding frame incorporating emergent themes from the data. Each article was double-coded.

**Results:**

The frequency of relevant articles rose and fell in line with policy debate events. Children were frequently characterised as victims of SHS, and SHS was associated with various health risks. Articles discussing legislation targeting SHS in private vehicles carrying children presented supportive arguments significantly more frequently than unsupportive arguments.

**Conclusions:**

The relatively positive representation of legislation prohibiting smoking in vehicles carrying children is favourable to policy advocates, and potentially indicative of likely public acceptance of legislation. Our findings support two lessons that public health advocates may consider: the utility of presenting children as a vulnerable target population, and the possibility of late surges in critical arguments preceding policy events.

## Background

In 2011, the British Medical Association called for all private vehicles to be added to existing bans on smoking in enclosed public spaces throughout the UK [1], highlighting the restrictive interior spaces in vehicles, the specific vulnerabilities to second-hand smoke of children and elderly people, and children’s lack of agency to refuse to share a vehicle with smokers [1]. The risks of second hand smoke (SHS) are increasingly well understood [2, 3], and SHS is estimated to account for more than 600,000 [4] of the six million tobacco-related deaths worldwide each year [5]. Bans on smoking in enclosed public spaces throughout the United Kingdom (UK) predominantly restrict non-smokers’ exposure to SHS to private homes and vehicles, and the private vehicle has been identified as potential focus of future legislation designed to further limit non-smokers’ exposure to SHS.

In 2014, members of the UK Parliament voted to add an amendment to the Children and Families Bill empowering the Government to introduce legislation prohibiting smoking in vehicles carrying children, and bans are expected to come into force in England and Wales in October 2015 [6, 7]. The Smoking (Children in Vehicles) (Scotland) Bill, which would prohibit smoking in vehicles carrying children, was introduced to the Scottish Parliament in December 2014 and, at the time of publishing, is under scrutiny of the Health and Sport Committee [8].

When conceiving and promoting public health policy, policymakers must take into account the interests and attitudes of the public to ensure that policies are appropriate and acceptable. Mass media are a key influence on the public’s awareness and understandings of issues. In their theory of agenda setting, McCombs and Shaw [9] describe how the mass media set the political agenda, influencing which topics occupy public awareness by determining how much coverage those issues receive, and where that coverage is situated. In addition to influencing which issues are on the political agenda, framing theory [10] suggests that the media construct frames that influence how those interviews are presented. Frames can incorporate definitions of problems, diagnoses of causes, and moral judgements about those causes and suggested solutions [11], and these elements of media representations influence audiences’ constructions of their own individual-level frames, in turn influencing their attitudes towards those problems, causes and proposed solutions [10].

Through agenda setting and framing processes, mass media coverage influences which issues the public are aware of, and what their attitudes towards those issues are; public attention towards an issue has been shown to correlate with media focus on that issue [12], and media frames have been shown to influence recipients’ appraisals and decision-making [13]. Media content has been found to influence public understandings of SHS, specifically [14]. Given the influence the media have over public understandings and attitudes, public health policy development and advocacy can benefit from understanding mass media representations of issues.

A key part of framing a problem is the construction of the affected groups, and therefore the population targeted by any suggested solutions, as constructions of groups can influence how audiences appraise solutions. Schneider and Ingram [15] suggest that policymakers may categorise target populations by two axes: power and social construction. In this typology, a group can be politically weak or powerful, and can be constructed either positively or negatively [15]. Children are a politically powerless, positively-constructed group that attract sympathy and, when characterised as a target group, potentially engender support for legislative solutions. In the realm of tobacco control legislation specifically, Freeman, Chapman and Storey [16] describe the need to protect vulnerable children as *‘an almost invincibly powerful sub-text’* (p.64) against which industry lobbyists are unwilling to argue.

In this study we examine a decade of UK newspaper reporting on issues surrounding children’s exposure to SHS, analysing the prominence given to different aspects of the issue, the representations of the problem(s), constructions of affected groups and appraisals of legislative solutions. We focus particularly on children’s exposure to SHS in vehicles, which recently became prominent in UK political debate. We anticipate that this study is the first quantitative content analysis of UK newsprint coverage of children and SHS.

## Methods

Twelve UK national newspapers and six Scottish national newspapers with high circulations [17] were selected to represent each national perspective. Using a typology employed in previous content analyses of UK newspapers [18–20], three different genres of newspaper were included to ensure that the sample represented a diverse range of readership profiles. Tabloid genre newspapers (*n* = 6) are printed in the tabloid format and tend to be sensationalistic and attract a predominantly working class, politically diverse readership. Middle-market tabloid newspapers (*n* = 4) are also in the tabloid format, but are more serious and attract predominantly right wing, middle class readers. Serious (*n* = 8) genre newspapers have traditionally been published in the broadsheet format, are serious and politically diverse with a broadly middle class readership. Table 1 lists the publications used by region and genre. The timeframe, beginning 1st January 2004 and ending 16th February 2014, allowed baseline measurement of news reporting prior to the implementation of smoke-free legislation in Scotland in 2006, and encompasses the vote in the House of Commons empowering the UK government to introduce legislation to prohibit smoking in private vehicles carrying children on 10th February 2014.

**Table 1:** Overview of articles by region, genre and publication

		All articles		Front page articles	
		*n*	%	*n*	%
UK (*n* = 221)					
Serious (*n* = 51)	Daily Telegraph	28	6.6	3	23.1
	Guardian	16	3.8	0	0
	Observer	7	1.7	1	7.7
Middle-market tabloid (*n* = 97)	Daily Mail	71	16.8	n/a^a^	n/a^a^
	Express	20	4.7	0	0
	Mail on Sunday	3	0.7	1	7.7
	Sunday Express	3	0.7	0	0
Tabloid (*n* = 73)	Mirror	50	11.8	0	0
	Daily Star	19	4.5	0	0
	Sunday Mirror	4	0.9	0	0
Scotland (*n* = 201)					
Serious (*n* = 138)	Scotsman	67	15.9	2	15.4
	The Herald	61	14.5	5	38.5
	The Sunday Herald	6	1.4	1	7.7
	Scotland on Sunday	4	0.9	0	0
Tabloid (*n* = 63)	Daily Record	57	13.5	0	0
	Sunday Mail	6	1.4	0	0
Total		422	100	13	100

^a^Nexis database entries for the Daily Mail do not list page numbers

Researchers searched the *Nexis* database with the term: “*smok! OR tobacco OR cig! OR second hand smok! OR passive smok!” AND “babies OR baby OR child! OR kid! OR infant! OR early years OR toddler! OR tot! OR parent! OR mum! OR dad! OR car! OR vehicle!*”. The search retrieved 1572 articles. Researchers read each article and removed 1150 that met any of the following exclusion criteria: article is from an Irish edition; article is from the TV guide, review, sports, travel, weather or readers’ letters sections; article is a duplicate of a previously-included articles; less than half of the article text is relevant to children and SHS. The exclusion criteria were chosen to ensure that the sample contained only news articles relevant to the issue of children and SHS. Following the application of the exclusion criteria, 422 articles remained.

Researchers developed a coding frame with which to record the relevant manifest content of the articles. An initial coding frame structure was established from a priori knowledge about the topic, including the following thematic categories: health risks to children; adults and primary carers; environment; policy responses; societal and cultural factors. To organically generate emergent themes, researchers read 100 randomly-selected articles, adding thematic codes to the thematic categories in the coding frame as they emerged. Further batches of 20 articles were read until no further novel codes emerged. Table 2 lists the thematic categories that comprised the final coding frame.

**Table 2:** Thematic codes by frequency

	Total	
	*n*	%
Health risks to children		
Mentions children as victims of SHS exposure	280	66.4
Mentions that SHS is related to children’s health	261	61.9
Mentions harms to foetuses from SHS during pregnancy	144	34.1
Mentions exposure-duration or concentration of SHS as health risk	109	25.8
Mentions later-life biological harms to children of SHS	72	17.1
Mentions behavioural harms to children of SHS	64	15.2
Mentions children as advocates against SHS	53	12.6
Mentions third-hand smoking as a harm to children	17	4.0
Adults and primary carers		
Mentions non-specified adults’ smoking behaviours	226	53.6
Mentions parents’ awareness of SHS and behaviour modification	120	28.4
Mentions poor parenting in relation to SHS and children	72	17.1
Mentions parents as unaware or lacking education about SHS	65	15.4
Mentions parental deprivation, lack of education or unhealthy lifestyles	62	14.7
Mentions mothers’ smoking (exc. during pregnancy)	51	12.1
Mentions parents’ awareness of SHS and no behaviour modification	49	11.6
Mentions fathers’ smoking	38	9.0
Mentions harms to mothers of smoking during pregnancy	31	7.4
Environment		
Mentions harms to children of SHS exposure in the home	143	33.9
Mentions harms of SHS exposure in vehicles to children	116	27.5
Mentions harms to children of SHS exposure in public places	35	8.3
Policy responses		
Mentions solutions for SHS other than legislation	142	33.7
Mentions arguments supporting prohibition of smoking in vehicles carrying children	100	23.7
Mentions arguments opposing prohibition of smoking in vehicles carrying children	73	17.3
Mentions consequences for children of the smoking ban in public places	63	14.9
Mentions a ban on smoking in public places to protect children from SHS	54	12.8
Mentions other policies limit children’s exposure to SHS	45	10.7
Mentions other countries’ policies to protect children from SHS	29	6.9
Societal and cultural factors		
Mentions anti-legislation stance (excluding smoke-free car legislation)	67	15.9
Mentions the costs of SHS to society (economy, health, loss of life etc.)	61	14.5
Mentions the vilification of, or and attacks on, smokers	47	11.1
Mentions de-normalisation of smoking	41	9.7

Researchers recorded the relevant manifest content of each article using the coding frame. Manifest content is that which is presented overtly and is quantifiable. It differs from latent content, which requires interpretive reading of meanings underlying surface-level data [21]. While latent content analysis is useful for nuanced qualitative analysis of representations of themes, manifest content analysis excels in allowing themes to be observed more broadly throughout a large sample, using quantitative analysis to identify trends and understand relationships between themes and other features of reporting. Each article was coded separately by two researchers, and each article could be coded for multiple themes. A coding definition document was updated throughout the coding process and used as a reference tool to ensure articles were coded consistently. In cases where researchers coded the same article differently, they discussed their interpretations of the text until consensus was reached. If the process of reaching consensus required that the definition of a code be altered, previously-coded articles were checked to ensure that their coding was consistent with the updated definition.

Data were analysed with Stata 11 [22]. Crosstabs and frequency tables were used to produce descriptive statistics. Spearman non-parametric correlation tests were used to measure the direction and significance of changes in the frequency of reporting over time. Paired t-tests were used to measure differences between two observations. Statistical significance is defined as *p* < 0.05.

Ethical approval for this project was granted by the University of Glasgow College of Medicine and Veterinary Science research ethics committee.

## Results

### Overview of sample

During the sample period of 1st January 2004 to 16th February 2014, 422 articles reporting on SHS and children were published within the 18 sample publications. Of those 422 articles, thirteen (3.1 %) were published on front pages. Table 1 lists the number of articles and front page articles by publication, genre and region.

Publications were separated into those distributed throughout the UK, including Scotland, (*n* = 10) and those distributed exclusively within Scotland (*n* = 6). These totals exclude two UK publications (the serious genre Sunday Telegraph and the tabloid genre Daily Star on Sunday) which printed no relevant articles and are not represented in the sample. More than half (*n* = 221, 51.0 %) of articles were printed in UK publications. Scottish publications printed 33.5 articles per publication, while UK publications printed 18.4, suggesting that issues related to SHS and children had a higher newsprint profile in Scotland compared to the UK as a whole.

Seven of the publications represented the serious genre, four middle-market tabloid and five tabloid. In absolute terms, serious genre articles were most frequent (*n* = 189, 44.8 %), followed by tabloid (*n* = 136, 32.2 %), while middle-market tabloid articles were least frequent (*n* = 97, 23.0 %). However, there was little difference in the average number of articles per publication; serious genre publications printed 23.6 per publication, middle-market tabloids printed 24.3, and tabloids printed 22.7.

### Articles reporting on SHS in vehicles carrying children

Nearly one third of articles (*n* = 129) reported on SHS in vehicles carrying children, either by discussing harms posed to children by SHS exposure in vehicles (*n* = 116, 27.5 %), or by mentioning arguments for or against legislation intended to reduce children’s exposure to SHS in vehicles (*n* = 105, 24.0 %). Per publication, middle-market tabloids (10.3) reported on SHS in vehicles carrying children more frequently than did serious (6.5) or tabloid (6.0) publications. Scottish and UK sources published the same number of articles per publication (7.2).

One quarter (*n* = 105) of articles mentioned arguments for or against legislation intended to reduce children’s exposure to SHS in vehicles. Supportive arguments (*n* = 100, 95.2 % of the 105 articles mentioning arguments for or against) were significantly (*p* < 0.000) more frequent than critical arguments (*n* = 73, 69.5 %). Two-thirds (*n* = 68, 64.8 %) of articles mentioning arguments reported both supportive and critical arguments. Thirty-two (64.8 %) articles exclusively mentioned supportive arguments, while five (4.8 %) exclusively mentioned critical arguments. The only year in which critical arguments (*n* = 19) outnumbered supportive arguments (*n* = 17) was 2014, but the whole year was not represented in the sample.

### Trends in reporting over time

**Fig. 1:**
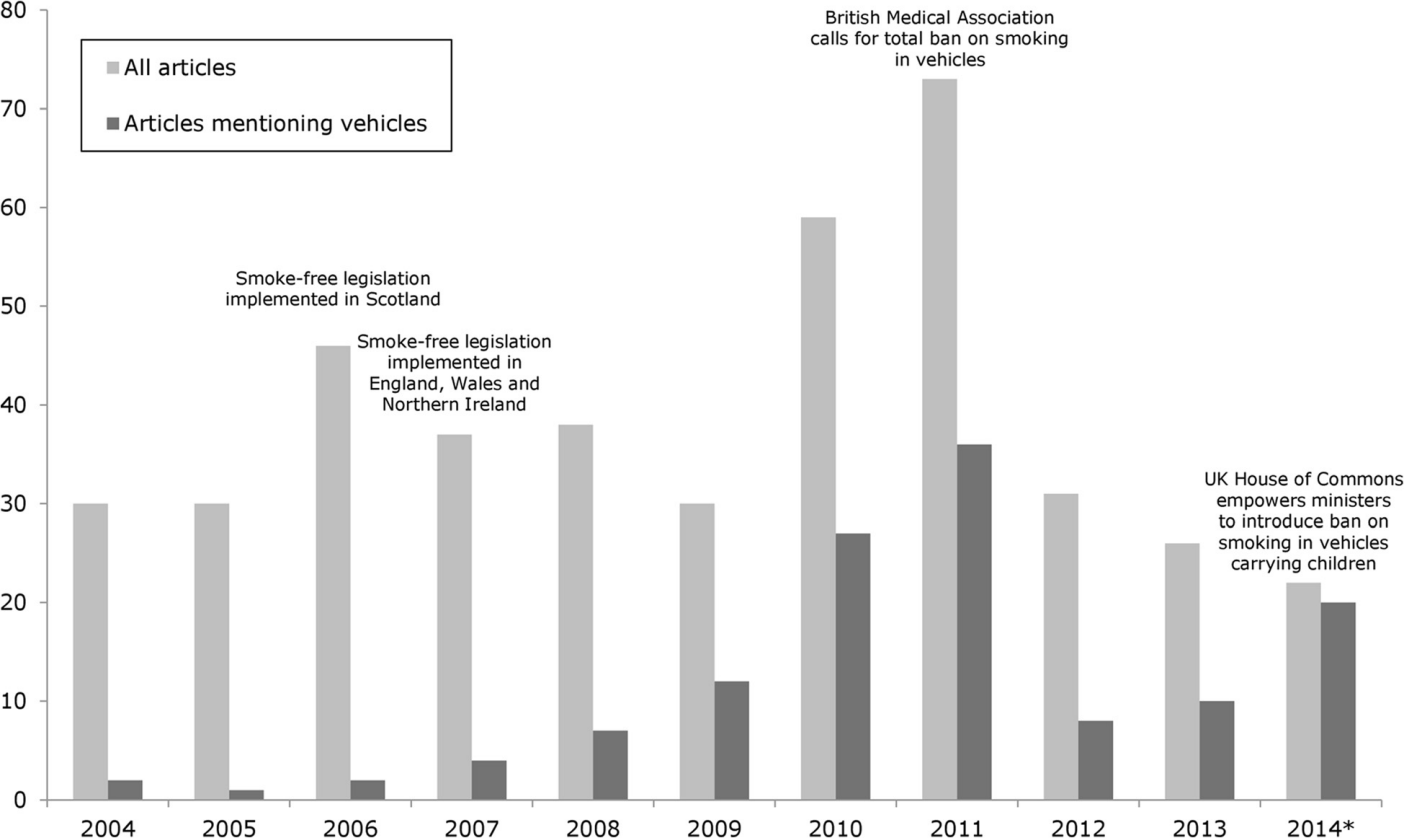
Frequency of articles by year of publication

There was a gentle, but non-significant overall increase in the frequency of articles per year, with a peak of 73 articles in 2011, largely related to the BMA’s call for a ban on smoking in all vehicles, including those not carrying children [1]. The frequency of articles mentioning SHS in vehicles carrying children (*n* = 129) increased significantly (p = 0.003) across the sample period, as did the proportion of the wider sample for which they accounted (*p* < 0.000), indicating that it became an increasingly prominent aspect of the topic of children and SHS. While only 22 relevant articles were published in 2014, this accounts only for a month and a half, in which the number of articles published per day (*n* = 0.5) was greater than in 2011 (*n* = 0.2). Figure 1 illustrates the frequency of relevant articles over time, and highlights the major policy events corresponding with peaks in reporting.

### Representations of threats to children

Articles frequently framed SHS as a general threat to children’s health (*n* = 261, 61.9 %). Specific types of harms mentioned included later-life biological harms, such as cancers (*n* = 72, 17.1 %), and behavioural harms, such as associations with depression (*n* = 64, 15.2 %). Two-thirds (*n* = 280) of articles used language characterising children as victims of SHS, and a quarter (*n* = 109) mentioned the roles of exposure duration and atmospheric concentration in SHS risks. Seventeen (4.0 %) articles mentioned third-hand smoking, while one third (*n* = 143) mentioned harms to children from SHS exposure in the home.

Despite being widely characterised as victims of SHS, children were not always portrayed as passive; 53 (12.6 %) articles mentioned that children play the role of advocates against SHS, either through direct attempts to dissuade adults from smoking, or indirectly in campaigners’ use of children’s experiences within advocacy strategies.

## Discussion

Reporting on issues related to children and SHS grew in frequency across the sample period, punctuated by policy debate events, and coverage was more frequent in Scottish publications than UK publications. The proportion of articles that were published on front pages (3.1 %) was smaller than that proportion in studies of newsprint coverage of the H1N1 outbreak (4.7 %) [23], the obesity epidemic (3.9 %) [24] and minimum unit pricing for alcohol (5.9 %) [25] employing similar methods. McCombs [26] highlights front page positioning as a cue that communicates a topic as highly salient, and our findings indicate that the topic of legislation to prohibit smoking in cars carrying children may have been lower on the news agenda than these other health issues, though the differences are not stark.

The issue of prohibiting smoking in private vehicles carrying children became increasingly prominent in newspaper news coverage over the sample period. Arguments in favour of legislation designed to prohibit smoking in vehicles carrying children were reported significantly more frequently than arguments against, suggesting a tone of coverage relatively favourable to legislation, although not as overwhelmingly so as in the Australian debate [16]. Articles largely identified SHS as a threat to children’s health and characterised children as victims, contributing to a frame sympathetic towards legislation designed to protect children [15, 16]. The focus on the protection of vulnerable children may have invoked the sub-text described by Freeman and colleagues [16], which may go some way to explaining the predominantly supportive coverage of the proposed legislation. A qualitative analysis of a subsample of the articles studied in this project found that children were characterised as in need of protection from smoking adults’ behaviours [27].

The only year in which critical arguments outnumbered supportive arguments was 2014, a period of frequent reporting in the weeks before and immediately following the vote in the House of Commons empowering the UK government to introduce legislation to prohibit smoking in private vehicles carrying children, and the lodging of the Smoking (Children in Vehicles) (Scotland) Bill in the Scottish Parliament. This late increase in critical arguments supports Harris and colleagues’ [28] recommendation that policy advocates should expect increased opposition in the final weeks preceding a policy event.

Legislation ensuring smoke-free indoor public spaces across the UK has been effective [29–32] and popular [33], and private vehicles carrying children have been identified as a next step in smoke-free legislation [1, 3, 34]. The rising profile of the issue across our sample period will be welcomed by advocates of the legislation and policy developers can draw confidence from the relatively positive representations of legislative solutions in the media, which could be an influence on, and indicator of, public reception. Advocates involved in tobacco control and public health in jurisdictions outside the UK may be able to apply the findings of this UK case study in planning future advocacy work, whether related to SHS exposure, tobacco control or broader public health issues.

Some limitations of this research should be considered. While quantitative content analysis allows the manifest content of large samples to be examined broadly, it is not suited to studying specific themes in detail. Further research could use qualitative analysis to explore specific aspects in greater depth, such as how different arguments are represented. A limitation inherent to content analysis is that claims about authors intentions and audiences’ interpretations cannot be made; complementary audience reception research could compare media representations with public perceptions of the issues. Finally, our exclusive focus on newsprint is at the expense of insight into representations within other media, which further research might incorporate.

## Conclusions

The issue of children’s exposure to SHS has become increasingly prominent in UK newspapers. The predominantly supportive representation of arguments about legislation prohibiting smoking in vehicles carrying children is a positive sign for advocates engaged in the debate, and may serve as encouragement for policymakers. Our findings echo recommendations from existing literature that communicating with the public about the harms of SHS can be more effective if messages focus on the vulnerability and powerlessness of children, and that advocates should be wary of, and prepared to offer rebuttals to, late surges in arguments opposing legislative change in the days preceding policy events.

## Abbreviations

SHS: Second-hand smoke
UK: United Kingdom
